# Orthopaedic residents demonstrate retention of point of care ultrasound knowledge after a brief educational session: a quasi experimental study

**DOI:** 10.1186/s12909-019-1916-0

**Published:** 2019-12-30

**Authors:** Samuel Larrivée, Robyn Rodger, Patricia Larouche, Jeff Leiter, Tomislav Jelic, Peter MacDonald

**Affiliations:** 1 Department of Surgery, Section of Orthopaedic Surgery, Rady Faculty of Health Sciences, University of Manitoba, AD4 – 820 Sherbrook St, Winnipeg, Manitoba R3A 1R9 Canada; 2 Department of Emergency Medicine, Rady Faculty of Health Sciences, University of Manitoba, Winnipeg, MB Canada

**Keywords:** Point of care ultrasound, Musculoskeletal ultrasound, Sonography, Resident education, Orthopaedic surgery, Online curriculum

## Abstract

**Background:**

Musculoskeletal point of care ultrasound (MSK POCUS) has many uses for orthopaedic surgeons, but orthopaedic trainees are rarely exposed to this modality. The purpose of this project was to assess the usefulness in clinical education of a newly implemented MSK POCUS course in an orthopaedic surgery program.

**Methods:**

An MSK POCUS course for orthopaedic surgery residents was developed by an interdisciplinary team. Online videos were created to be viewed by residents prior to a half-day long practical course. An online survey covering the level of training of the resident and their previous use of ultrasound (total hours) was completed by the participants prior to the course. Resident’s knowledge acquisition was measured with written pre-course, same-day post-course and six-month follow-up tests. Residents were also scored on a practical shoulder examination immediately after the course and at six-month follow-up. Changes in test scores between time points were evaluated using Wilcoxon signed-rank tests.

**Results:**

Ten orthopaedic surgery residents underwent the MSK POCUS curriculum. Pre-course interest in MSK-POCUS was moderate (65%) and prior exposure was low (1.5 h mean total experience). Written test scores improved significantly from 50.7 ± 17.0% before to 84.0 ± 10.7% immediately after the course (*p* < 0.001) and suffered no significant drop at 6 months (score 75.0 ± 8.7%; *p* = 0.303). Average post-course practical exam score was 78.8 ± 3.1% and decreased to 66.2 ± 11.3% at 6 months (*p* = 0.012). Residents significantly improved their subjective comfort level with all aspects of ultrasound use at 6 months (*p* = 0.007–0.018) but did not significantly increase clinical usage frequency.

**Conclusion:**

An MSK POCUS curriculum was successfully developed and implemented using an interdisciplinary approach. The course succeeded in improving the residents’ knowledge, skills, and comfort with MSK POCUS. This improvement was maintained at 6 months on the written test but did not result in higher frequency of use by the residents.

## Background

“Ultrasound is an extension of the stethoscope” is an oft-cited catchphrase that is rapidly becoming more accurate as point of care ultrasound (POCUS) becomes more readily accessible in the world of medicine. Historically an imaging modality limited to radiology departments and daylight hours, the recent evolution of ultrasound has led to its usage in operating rooms, in emergency departments, on wards and in offices by a vast spectrum of clinicians [1]. Arguably the least invasive imaging modality currently available, with significant portability, relative ease of use and relevance to almost any medical specialty, it’s no wonder ultrasound has become a mainstay of bedside diagnosis and procedural guidance [2].

In addition to being a useful adjunct to a physical exam and procedural skills for generalist medical practitioners, there are also numerous, more specific POCUS skills that can be readily applied in specialist and subspecialist settings. One such modality is that of musculoskeletal ultrasound (MSK POCUS). Sonography enables the diagnosis of many musculoskeletal pathologies including muscle sprains and tears, ligament injuries, tendon inflammation and ruptures and fractures [3–5]. From a treatment perspective, ultrasound helps to guide joint injections and aspirations, and confirm adequate reduction of fractures and dislocations immediately post-reduction [4, 6]. When used to guide forearm fracture reduction in comparison to the traditional blind method, MSK POCUS has been shown to increase the certainty [7] and success rate of closed reductions [7, 8], decrease the number of attempts and decrease the need for operative intervention [8, 9].

A further benefit of MSK POCUS is its ability to decrease the more expensive, time-consuming use of MRI for diagnostic purposes. In addition to not being subject to any contraindications, ultrasound allows immediate bilateral comparisons, differentiates fluids from solids more accurately than MRI and allows for real-time dynamic examination [10]. In the hands of a competent orthopaedic surgeon, an office-based ultrasound of the shoulder can provide a definitive diagnosis of a rotator cuff tear. When surgeons use POCUS plus physical exam, the diagnosis is more accurate than radiologist-read MRI, when intra-operative findings are used as the gold standard [11]. Thus office-based shoulder ultrasound allows definitive diagnosis of rotator cuff injury to happen with less delay and in a single setting, and MRI to be preserved for evaluation of more complex injuries [12].

Given ultrasound’s numerous advantages, it has become commonplace for medical schools to introduce learners to POCUS and many residency programs, such as emergency medicine, general surgery and anesthesia now involve extensive POCUS training. In orthopaedic surgery residencies in Canada, however, ultrasound skills are not a requirement for board certification by the Royal College of Physicians and Surgeons of Canada [13]. Furthermore, no published evidence of MSK POCUS being required in other orthopaedic surgery training programs elsewhere could be found. As a result, MSK POCUS has yet to become a skill commonly acquired by orthopaedic residents. Similar gaps in ultrasound training have also been noted in family medicine [14] and internal medicine [15].

In reviewing the literature surrounding point of care ultrasound education, it is noted that simulation and web-based learning are gaining ground as excellent, evidence-based adjuncts to clinical POCUS training and hands-on sessions [16]. By allowing learners to improve ultrasound skills and knowledge prior to attempting to use the modality in the clinical setting, patient safety and comfort is notably improved [17] and procedural complications are reduced [18]. In the orthopedics world specifically, Piposar et al. [19] found that orthopedics residents significantly improved their ability to assess ankle tendons with ultrasound after a multimedia tutorial alone. Building upon these concepts, a blended learning model has been described as one that combines multimedia-based learning tools, such as online modules, with simulation and hands on sessions with real or standardized patients [16]. This blended approach is thought to improve overall learner proficiency when compared to more traditional apprenticeship models [20]. Further reinforcing this approach is a publication by Finnoff et al. [21], in which a group of physical medicine and rehabilitation residents (PM&R) completed a blended learning MSK POCUS course and demonstrated marked improvement in ultrasound-related general knowledge image acquisition and injury assessment.

Given the many procedural, diagnostic and cost-saving benefits alluded to previously, it stands to reason that in the future, MSK POCUS will become a cornerstone of managing orthopedics patients, both in the acute setting and in the office. To better understand how these skills can best be acquired by orthopedics residents, a study was designed with the purpose of assessing the utility of administering a general MSK POCUS curriculum to orthopedics residents using a blended learning model. The hypothesis was that a brief MSK POCUS course using a blended learning model would improve orthopaedic surgery residents’ performance in ultrasound guided diagnosis of musculoskeletal injury, improve confidence in reduction of fractures, and improve ability to perform difficult joint injections and aspirations.

## Methods

### Curriculum development

An MSK ultrasound curriculum was developed by a multidisciplinary team, which was comprised of a Royal College trained attending physician in emergency medicine with a fellowship in point of care ultrasound, an attending pediatric orthopaedic surgeon, an orthopaedic surgery resident and an emergency medicine resident. The course drew inspiration by the one developed by Finnoff, Smith [21], but was heavily modified to target ultrasound skills deemed relevant to orthopaedic surgery based on a literature review and the combined clinical experience of the team. Given the busyness of the teaching curriculum in the orthopaedic surgery residency, it was decided that the online portion of the course should be short, and the practical portion should be done in one half-day of teaching. The final curriculum consisted of:

- A two-hour online course completed independently by the learners prior to the practical session. Content included a review of ultrasonography basics (physics, knobology), followed by an overview of sonographic appearance of normal MSK tissues (bone, tendon, ligament, muscle and large joints) as well as ultrasound technique for generating the relevant images. The course concluded with an overview of sonographic appearance of pathological MSK tissues (long bone fractures, ligament and tendon tears, bursitis, rotator cuff tears) and common pitfalls in point of care MSK ultrasound.
- A four-hour practical session administered by the attending emergency medicine physician with ultrasound fellowship training and an orthopaedic surgery resident and emergency medicine resident. This session covered practical demonstrations of the examination of the shoulder (including rotator cuff), elbow and ankle joints and included ample hands-on practice time for participants. There was also training time dedicated to ultrasound-guided musculoskeletal injections and aspirations and ultrasound-guided fracture reductions, both of which were taught using models. (See Additional file 1)

### Study design

Approval was obtained from the appropriate ethics board. Given the small size of the orthopaedic surgery residency program being studied, a quasi-experimental study design was selected, and a pre-test post-test approach was utilized. It was determined that all orthopaedic surgery residents would benefit from MSK ultrasound training and should be exposed to the proposed curriculum, therefore no control group was used.

Inclusion criteria included being an orthopaedic surgery resident completing their residency training at the study site. Any resident in poor standing with the program, having less than 6 months left before graduation, or on extended leave was to be excluded.

Recruitment of participants was done by adding the MSK POCUS curriculum to program-scheduled academic days with the support of the local program directors. All of the orthopaedic surgery residents were required to attend as a component of their residency education, but they were given the option of declining participation in pre and post testing. Informed consent was provided by all participants. Participants were provided with a monetary compensation of twenty dollars per assessment.

### Outcome measures

Prior to the intervention (ultrasound course), all participants completed a pre-test via password-protected online survey examining theoretical knowledge of MSK ultrasound, including identifying pathology and using ultrasound technology (See Additional file 2). An online survey assessing previous exposure to ultrasound (in hours), setting of the exposure, number of times used in the last 6 months and comfort performing the different aspects of the MSK POCUS exam was also administered at this time.

After completing both the online and practical components of the curriculum, the initial post-test was administered to all participants. Post-testing entailed completion of the previously detailed online exam administered as the pre-test, as well as a practical exam component. These assessments were completed immediately after the practical session. The practical exam included basic ultrasound usage and a complete shoulder assessment, including the rotator cuff and was designed to be used as a benchmark for ultrasound competence both initially and over time (See Additional file 3 for practical exam marking scheme). Satisfaction with different aspects of the course was also assessed with an online survey.

Six months post intervention, both the online and the practical exam were administered by the same members of the research team. Participants were allowed to review the online material and steps of the practical examination at their leisure before the assessment. The survey on participants use and comfort performing MSK POCUS in the previous 6 months was also re-administered at that time. (See Additional file 4 for clinical use survey).

### Data analysis

All statistical analyses were tested with a 0.05 threshold for significance and performed on SPSS statistics v22.0 ed. 64 bits for Windows (IBM, Armonk). Descriptive statistics including frequencies and means with standard deviation were calculated. Given the low number of participants, non-parametric Wilcoxon Signed-Rank tests were used to compare between time points. As the written test was assessed at three time points, a Bonferroni correction was applied for multiple Wilcoxon comparisons on this variable. Power calculations were performed using G*Power version 3.1.9.2 for Windows using data available from a similar project performed by Finnoff et al. [21]. With a power set a 80% and 0.05 threshold for statistical significance, four participants were required to obtain a significant improvement on a written test on ultrasound knowledge.

## Results

### Participants

**Fig. 1:**
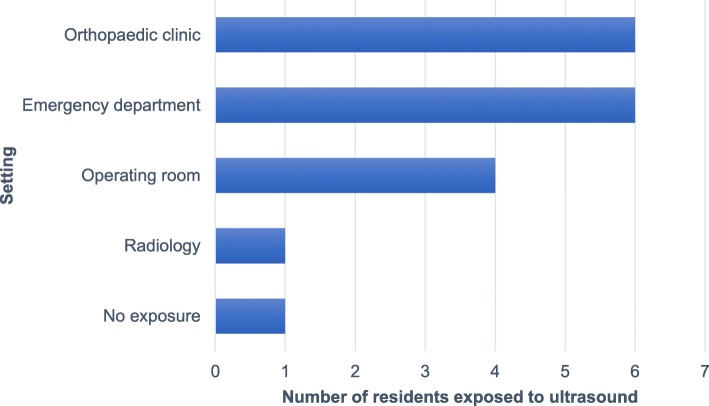
Residents’ previous exposure to ultrasound by clinical setting. For each setting, the number of residents who have mentioned being exposed to MSK ultrasound is shown

Ten residents participated in the online and practical ultrasound course and were recruited for the study. At the time, six residents were in their senior years (Postgraduate year 3 or more) and four were in their junior residency years (PGY 1 or 2). Prior to the course, interest in MSK POCUS was rated at 6.5 ± 3.6 out of 10 on a Numeric Assessment Scale (NAS). Exposure to ultrasound was minimal, at a mean of 1.5 ± 1.5 h in medical school and previous residency years. Residents have been mostly exposed to MSK POCUS in the orthopaedic sports clinic and the emergency department, followed by spine surgery operating room, and the radiology rotation (See Fig. 1). Each participant filled the pre-course questionnaires before getting access to the online tutorials, but two residents omitted to watch the material in time for the practical session. All of them were present for the whole duration of the practical session. Follow-up questionnaires and tests were completed at the required interval by every resident.

### Course appreciation

The course was rated of high quality by the residents, with an average rating of 8.7/10 ± 0.82 for the overall quality. Other aspects of the course fared similarly, with quality of the online videos rated at 8.7/10 ± 0.87 and the quality of the practical session rated at 8.8/10 ± 1.37. The length of the course was rated as adequate by most residents, with six saying that the length of the online videos was “Just about right”, and all residents agreeing that the length of the practical session was also “Just about right”. Sections that gathered 50% or less approval rate were the video sessions describing the abnormal pathologies seen on ultrasound, and the anatomy of the elbow and ankle (See Table 1). Free text comments left by the residents also attested to an interest in learning more about ultrasound guided nerve blocks and hip joint aspiration.

**Table 1:** Appreciation of curriculum length by residents

Online portion										
	Total length	Ultrasound physics	Setting up	Normal anatomy	Pathological anatomy	Shoulder anatomy	Elbow anatomy	Knee anatomy	Ankle anatomy	Total length
Far too much	1	0	0	0	0	0	0	0	0	0
Too much	2	0	0	1	1	1	1	1	1	0
About right	6	9	9	6	5	8	5	7	5	10
Too little	0	0	0	2	3	0	3	1	3	0
Far too little	0	0	0	0	0	0	0	0	0	0
N/A	1	1	1	1	1	1	1	1	1	0
Practical portion										
	Setting up	Shoulder exam	Elbow exam	Knee exam	Ankle exam	Joint inj/asp	Fracture reduction	Practice time		
Far too much	0	0	0	0	0	0	0	0		
Too much	0	1	0	0	0	0	0	0		
About right	9	9	7	9	8	8	8	10		
Too little	1	0	2	1	2	2	2	0		
Far too little	0	0	1	0	0	0	0	0		
N/A	0	0	0	0	0	0	0	0		

For each aspect of the course, the number of residents answering each item is displayed

Eight residents answered they were very likely to recommend the course to other residents. However only three said they were “very likely” to incorporate MSK POCUS in their future practice, while six said they were “somewhat likely” to include it.

### Retention of knowledge and skills

**Fig. 2:**
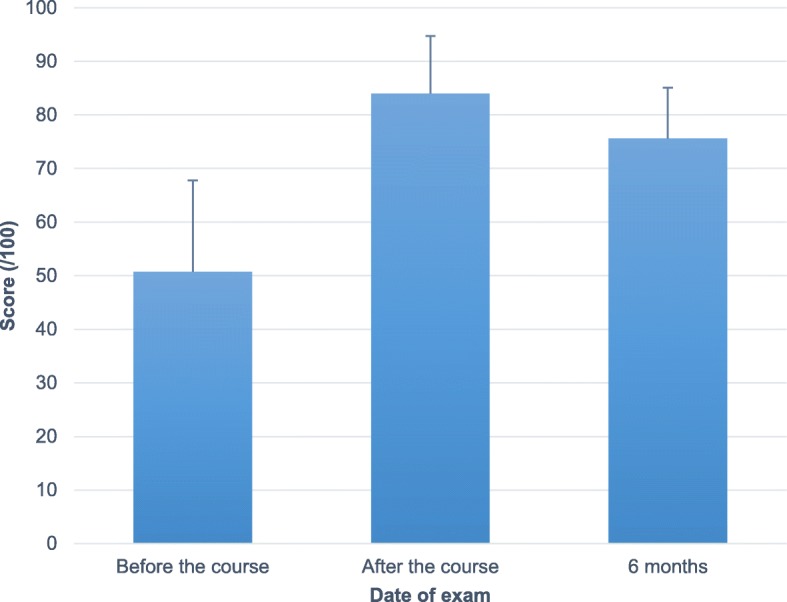
Written ultrasound examination scores. The average score (in percentage) and standard deviation of the written ultrasound examination are presented for the three timepoints

The average score on the written test was 50.7% ± 5.4% before taking the online and practical course. This score improved to 84.0% ± 3.4% immediately after the practical portion of the course. At 6 months, the average mark was 75.6% ± 3.0%. (See Fig. 2). Using pairwise Wilcoxon tests, the scores after the course and at 6 months both presented statistical improvement from the pre-course score (*p* = 0.005 and *p* = 0.008 respectively). This remained significant after Bonferroni correction. The difference in scores between the post-course test and six-month test was not statistically significant (*p* = 0.075).

**Fig. 3:**
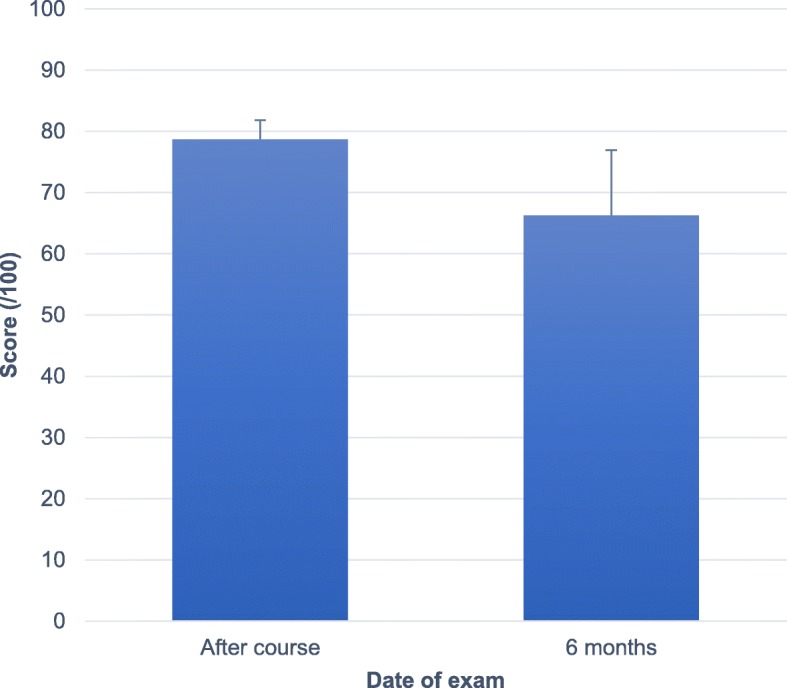
Practical shoulder exam scores. The average score (in percentage) and standard deviation of the practical ultrasound test are presented for the two timepoints following the course

Following the practical portion of the course, the residents’ average score at the graded shoulder ultrasound examination was 78.7% ± 3.1%. At the six-month examination, the average score had dropped significantly to 66.3% ± 10.7% (*p* = 0.018) (See Fig. 3).

### Comfort and use of ultrasound

**Fig. 4:**
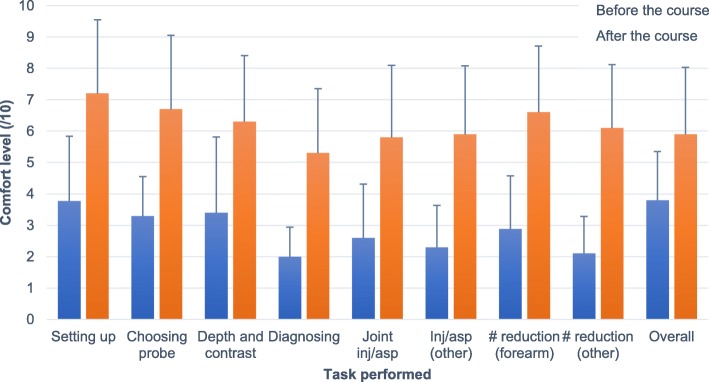
Residents’ comfort level in the 6 months before and after the course. Subjective comfort level (NAS) of the residents while performing ultrasound tasks in the 6 months preceding (blue) and the 6 months following (orange) the course. Abbreviations: inj = injection, asp = aspiration, # = fracture

Six months following the course, the residents’ comfort while performing MSK POCUS significantly improved from 3.80 ± 1.5/10 to 5.90 ± 2.1/10 on the NAS (*p* = 0.005). This held true for all aspects of the ultrasound examination evaluated (*p* = 0.005–0.011, see Fig. 4). However, the number of times the ultrasound was reported being used by the residents in clinical duties did not improve in the 6 months following the course when compared to the 6 months preceding it (0.9 ± 2.0 to 6.5  ±14.9 times used, *p* = 0.279). Taking a closer look at the individual numbers, five residents never used the ultrasound prior to the course and this did not change after the course. Three residents had never used it and started using after the course, one of whom recalled using ultrasound approximately 48 times in the 6 months following the session. Two residents described previous use of ultrasound, one of whom increased his use after the course while the other did not use ultrasound after the session. Testing for differences in practical or written scores between residents who increased their use and those who did not following the intervention did not reach statistical significance.

## Discussion

An MSK POCUS curriculum for orthopaedic residents was successfully developed and implemented into the orthopaedic training program. The development drew inspiration from the curriculum developed by Finnoff et al. [21] for PM&R residents. However, our ultrasound curriculum is significantly shorter and less extensive than that of Finnoff et al. [21]. The decision was made during the development of teaching the basics and concepts, and leaving the residents own interest guide their self-learning if they wanted to develop their knowledge further. This decision was rewarded by the residents’ good overall rating of the quality of the course, which was similar to the ratings seen in Finnoff et al. [21]‘s study. The residents also mostly described the course length as adequate but requested that more time be spent describing abnormal pathologies and covering ultrasound guided nerve blocks and hip aspirations. It was also hypothesized that orthopaedic residents already had more extensive knowledge of musculoskeletal anatomy and a better sense of the tridimensional spatial orientation of body, requiring less additional training in these areas. Our positive outcome results seem to support this assertion.

Interest towards learning ultrasound was lower in our study than that of Finnoff et al. [21], where average interest in learning MSK POCUS was 9.0/10. This discrepancy could be explained by the orthopaedic residents limited prior exposure to MSK POCUS, or by them not being aware of the different practical uses for orthopaedic surgeons. In contrast, the interest of physiatrist in using MSK POCUS as a means of evaluating pathologic soft tissue structures and guiding therapeutic procedures is well documented [21].

The orthopaedic residents’ 65% improvement in written test scores parallels the 67% increase seen in Finnoff et al. [21]‘s study. Despite a different written exam in the two studies, this demonstrates that both programs were able to improve significantly their participants’ knowledge of MSK POCUS. There was no significant drop in our residents’ written test score at 6 months, meaning the knowledge was maintained despite no further training in MSK POCUS. It is however unclear if residents reviewed their ultrasound knowledge prior to the six-month assessment. To our knowledge, no other study has assessed MSK POCUS knowledge retention at 6 months. Finnoff, Smith [21] also documented a 237% increase in practical scores following the course. We consciously elected against pre-testing ultrasound practical ability as it was felt to be a futile endeavour before the learners knew how the ultrasound machine functioned. However, our average score of 78.7% on the practical post-course shoulder examination was similar to the 73.3% score obtained by the participants in Finnoff et al. [21]‘s study. Despite using a different practical test, these results show residents were able to acquire substantial practical skills following our short curriculum. Unfortunately, these skills were not maintained at 6 months. This is likely related to a low number of opportunities to use ultrasound clinically in a busy orthopaedic surgery residency. Few rotations are amenable to ultrasound use, and only one consultant in our centre routinely uses ultrasound for clinical diagnosis of rotator cuff tears. Busy calls and minimal practice might also have discouraged residents from using it in the emergency department for diagnosis of soft-tissue injury, guidance of aspirations, and reduction of fractures. These assumptions are strengthened by the fact that no significant increase in use was reported at the six-month mark.

Residents’ subjective comfort level following the course improve significantly at 6 months for all aspects of ultrasound use. While no study reported on a similar outcome at 6 months, Piposar et al. [19] reported improved subjective comfort level of 15 orthopaedic residents after an online tutorial on ankle ultrasonography. Their comfort level improved from 2.3 to 6.8 on a 10 points visual analog scale, similar to our improvement from 3.8 to 5.9 out of 10. Their residents also had an accuracy of about 90% to identify and measures ankle tendons on a cadaver. Seventy-one percent of their orthopaedic residents answered that they were likely to use ultrasound in their future practice, while 90% of our residents were “likely” or “very likely” to consider using ultrasound in their career. This shows that the curriculum was found valuable by the participants.

Unfortunately, the intervention did not result in a significant increase in ultrasound use in the 6 months following the course. The possible reasons for this result are probably similar to the reasons previously suggested to explain the decrease in practical test scores: low number of opportunities, busy practices, time pressures, and variable interest. Availability and cost of the ultrasound machines on call was likely not a limiting factor, as multiple devices are available to physicians in the emergency departments frequented by the orthopedic residents. However, only one clinic, the sports orthopedic surgery clinic, has an available ultrasound device, limiting the opportunities for residents on other rotations. It is interesting to note that four residents increased their frequency of use following the session. The low number of participants might explain why this result was not significant. Applied to a larger cohort, this would result in a noteworthy number of orthopaedic residents using ultrasound as a result of following a short online and practical course. Similarly, multiple shoulder surgeons have implemented ultrasound in their clinical practice despite no or minimal prior experience. These studies highlighted results similar or better to radiologists at diagnosing rotator cuff tears when ultrasound was done as part of the patient’s clinical evaluation in conjunction with the history and physical exam (sensitivity 94–96.2%, specificity 84.6–97%, positive predictive value 92.9–96.6%, negative predictive value 79.5–95.3% and accuracy 89.5–95.8%). The results were, however, highly dependent on the operator experience and number of ultrasounds performed [11, 12, 22, 23].

Strengths of this study include a high participation rate from the residents (10 out of all 12 residents), no drop-outs or loss to follow-ups, and the follow-up of residents’ knowledge retention at 6 months. Limitations of this study include the use of mostly subjective questionnaires, scoring of the practical exam by resident evaluators without saving the images or confirmation by a trained radiologist. The low number of participants is also a significant limitation of the study. As there were only twelve orthopedic residents in the author’s institution, the number could have been improved by including other orthopaedic surgery programs in the study. There are however geographical and institutional barriers in doing so. Furthermore, sample size calculations performed prior to the start of study showed that only four participants were required to detect as significant change in a written ultrasound test. Future iterations of the curriculum will include a section on ultrasound-guided nerve blocks, as well as more practice on real patients with pathologies to address the previously highlighted shortcomings.

## Conclusion

An MSK POCUS curriculum for orthopaedic residents was successfully developed and implemented using an interdisciplinary approach. It was seen as a valuable learning experience by residents and improved their knowledge and comfort level of the procedure. This improvement was maintained at 6 months on the written test, but did not result in higher frequency of use by the residents. It is our opinion that MSK POCUS proficiency should be included in the competency-based curriculum for orthopaedic residents. Further studies on the subject should try to document accuracy of residents for the diagnosis of MSK pathologies by ultrasound, as well as its effect on patients’ treatment pathways and cost-benefit analyses.

## Supplementary information

**Additional file 1.** Components of the MSK-US course. Bullet point description of the areas covered by the MSK POCUS course developed for this project. This list was used to create the online curriculum and practical session.

**Additional file 2.** Musculoskeletal ultrasound – Written test. Printout of the online musculoskeletal ultrasound written test given to the participants before the course and at each follow-up endpoint.

**Additional file 3.** Practical exam evaluation grid. The correction grid used by the evaluators to rate the participants’ performance while performing a diagnostic examination of the shoulder.

**Additional file 4.** Use of ultrasound in clinical setting. Printout of the online survey given to participants before the course and after 6 months. It evaluates their subjective comfort performing the different aspects of the MSK POCUS examination, as well as their frequency of use in the last 6 months.

## Data Availability

The datasets generated and analyzed during the current study are not publicly available due to the small number of participants and possible identification by association but are available from the corresponding author on reasonable request.

## Abbreviations

MSK: Musculoskeletal
POCUS: Point of care ultrasound
