# Supplementary material for: Orthopaedic residents demonstrate retention of point of care ultrasound knowledge after a brief educational session: a quasi experimental study
Source: BMC Med Educ. 2019 Dec 30;19:474. doi: 10.1186/s12909-019-1916-0 (PMC6937626; doi:10.1186/s12909-019-1916-0)
Supplement: Supplementary file 1 — Additional file 1. Components of the MSK-US course. Bullet point description of the areas covered by the MSK POCUS course developed for this project. This list was used to create the online curriculum and practical session. [file 12909_2019_1916_MOESM1_ESM.pdf]

UNIVERSITY OF MANITOBA

Department of Surgery – Section of Orthopaedic Surgery
AD-420 720 McDermot Ave Winnipeg Manitoba R3E 0T3
P (204) 787-1219   F (204) 787-2460

COMPONENTS OF THE MSK-US COURSE

Project Title: Usefulness of a Musculoskeletal Ultrasound Course in an Orthopedic Surgery Residency Training program

1) Online preparatory videos (1-2 hours)
   a) General ultrasound theory
   b) Setting and using the ultrasound machine
      i) Starting the machine
      ii) Selecting the appropriate probe
      iii) Adjusting depth and contrast
   c) Normal and pathological sonographic appearances of MSK structures
      i) Bones
      ii) Muscles
      iii) Tendons
      iv) Ligaments
      v) Bursae
      vi) Nerves
   d) Ultrasound anatomy of different regions of the MSK system
      i) Shoulder
      ii) Elbow
      iii) Knee
      iv) Ankle
2) Practical session (4-6 hours)
   a) Setting up and using the ultrasound machine
   b) The shoulder
      i) General exam
      ii) Biceps tendinopathy and rupture
      iii) Rotator cuff tendinopathy and rupture
      iv) Acromio-clavicular joint
   c) The elbow
      i) General exam
      ii) Distal biceps tendon rupture
   d) The knee
      i) General exam
      ii) Patellar tendon and quadriceps tendon
   e) The ankle
      i) General exam
      ii) Achilles tendon rupture
   f) Diagnosis of joint effusions

UNIVERSITY OF MANITOBA

Department of Surgery – Section of Orthopaedic Surgery
AD-420 720 McDermot Ave Winnipeg Manitoba R3E 0T3
P (204) 787-1219 F (204) 787-2460

g) MSK injections
    i) Shoulder
        (1) GH joint
        (2) Subacromial bursae
    ii) Foot and Ankle
        (1) Ankle joint
        (2) Subtalar joint
h) Reductions
    i) Distal radius fracture
    ii) Both bone forearm fracture
i) Practice with the ultrasound machine (after each section)
