# Supplementary material for: Orthopaedic residents demonstrate retention of point of care ultrasound knowledge after a brief educational session: a quasi experimental study
Source: BMC Med Educ. 2019 Dec 30;19:474. doi: 10.1186/s12909-019-1916-0 (PMC6937626; doi:10.1186/s12909-019-1916-0)
Supplement: Supplementary file 2 — Additional file 2. Musculoskeletal ultrasound – Written test. Printout of the online musculoskeletal ultrasound written test given to the participants before the course and at each follow-up endpoint. [file 12909_2019_1916_MOESM2_ESM.pdf]

UNIVERSITY OF MANITOBA

Musculoskeletal ultrasound - Written test

Written test

Thank you for participating in this research study. The goal is to assess of the effectiveness of a musculoskeletal ultrasound course in clinical education. Your answers to the following questions will help us assess how well residents can retain key concepts of the course.
All answers are strictly confidential.

Version date: June 2nd, 2017

* 1. Enter your participant number

* 2. Please indicate for which evaluation you are filling the survey:

- First evaluation (before the course)
- After the course
- Six (6) months follow-up
- Twelve (12) months follow-up

Beginning of the test

---

* 3. Fill in the blanks: The ____ probe is ideal for imaging superficial structures because of its _____ frequency and high resolution

- Curvilinear, high
- Linear, low
- Curvilinear, low
- Linear, high

* 4. Ligaments exhibit which type of artifact on ultrasound?

- Shadowing
- Reverberation
- Anisotropy
- Side lobe

* 5. A simple joint effusions will appear as:

- Isoechoic
- Hyperechoic
- Anechoic
- Hypoechoic

* 6. Fill in the blanks: Bone will appear as _____, with ______ posteriorly.

- Hypoechoic, shadowing
- Hyperechoic, shadowing
- Hypoechoic, acoustic enhancement
- Hyperechoic, acoustic enhancement

* 7. When scanning for fractures with ultrasound, which statement is**true**?

- Assess the bone in one plane
- A false positive for fractures on ultrasound is a growth plate
- A minimal cortical disruption of 5 mm can be detected
- The presence of a hematoma is not a sensitive sign for a fracture on ultrasound

* 8. How can anisotropy be differentiated from true tendon pathology?

- Apply pressure
- Rotate the probe 90°
- Angle the probe so that it is perpendicular to the area of concern
- Doppler

* 9. Which image demonstrates a fracture?

A

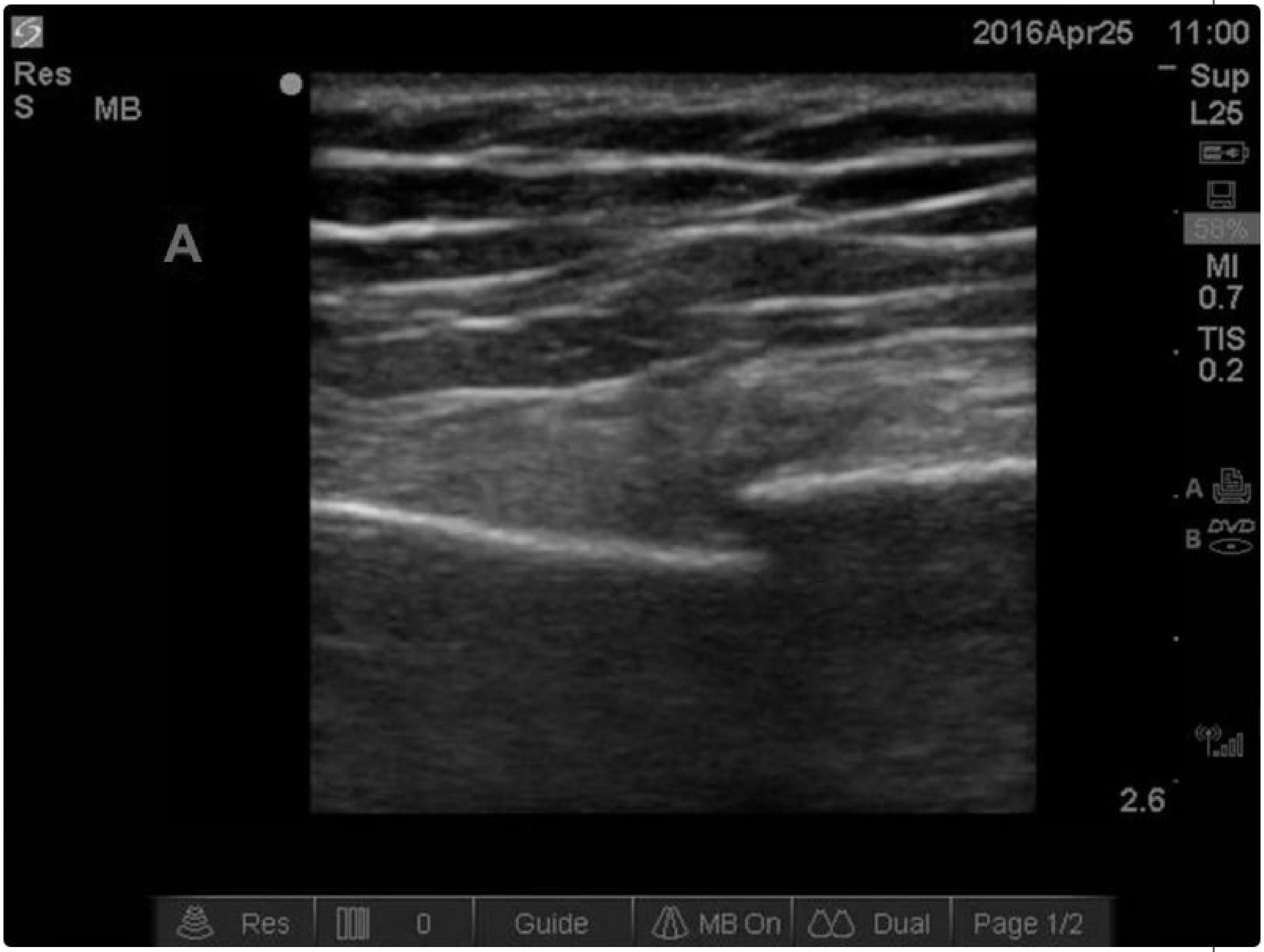

B

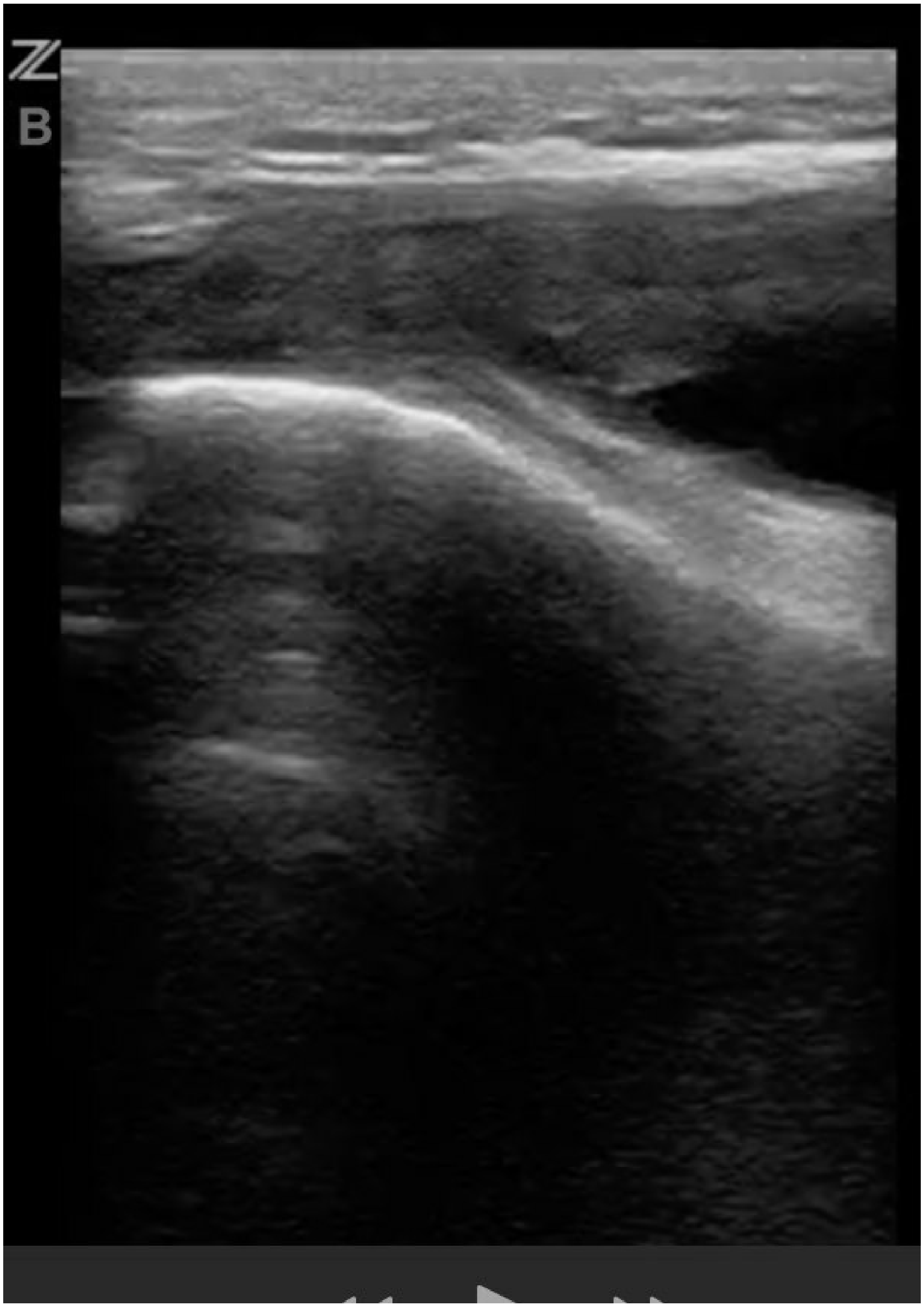

C

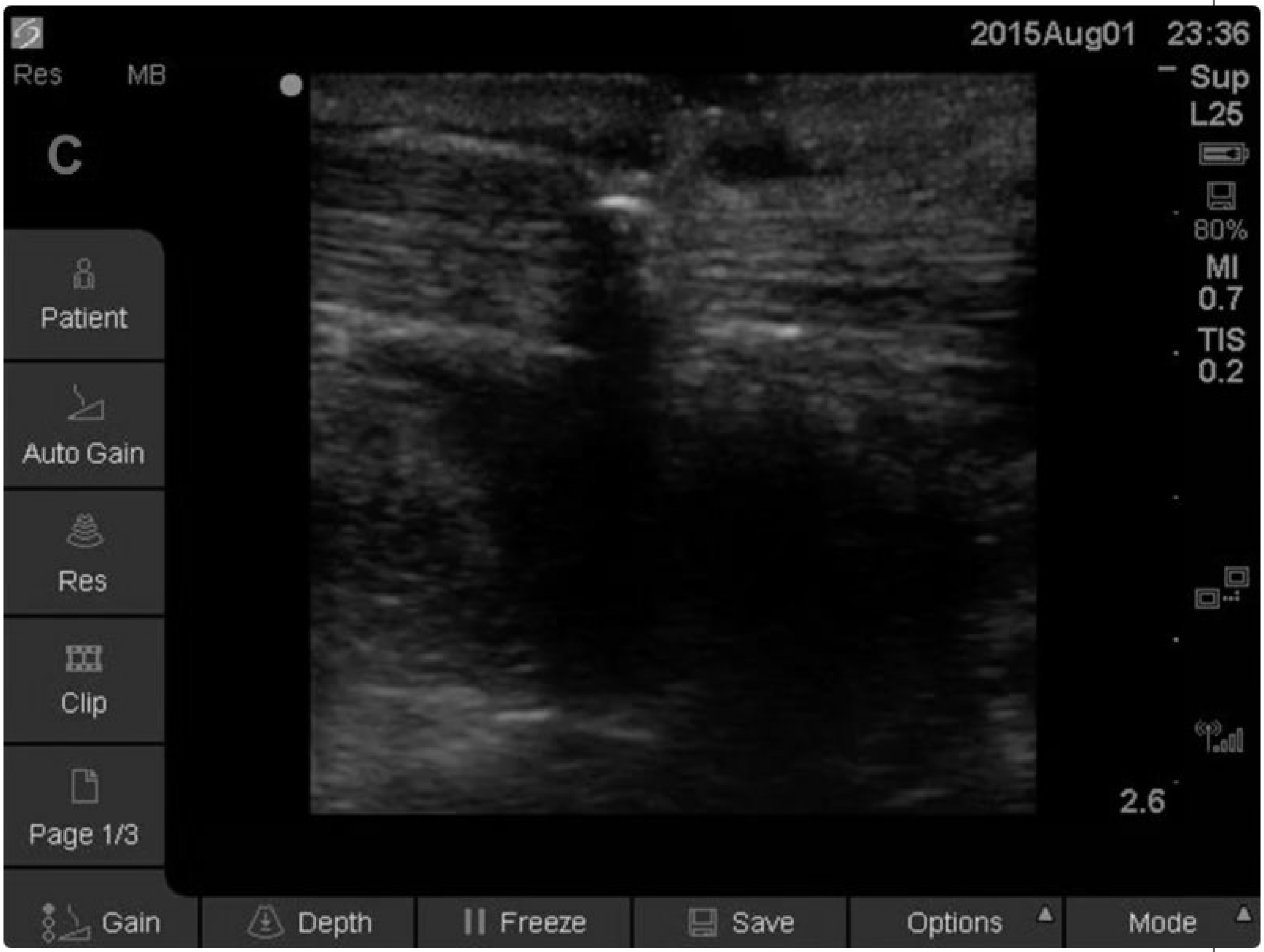
2015Aug01 23:36
Res MB
C
Sup
L25
80%
MI
0.7
TIS
0.2
Patient
Auto Gain
Res
Clip
Page 1/3
2.6
Gain
Depth
Freeze
Save
Options
Mode

D

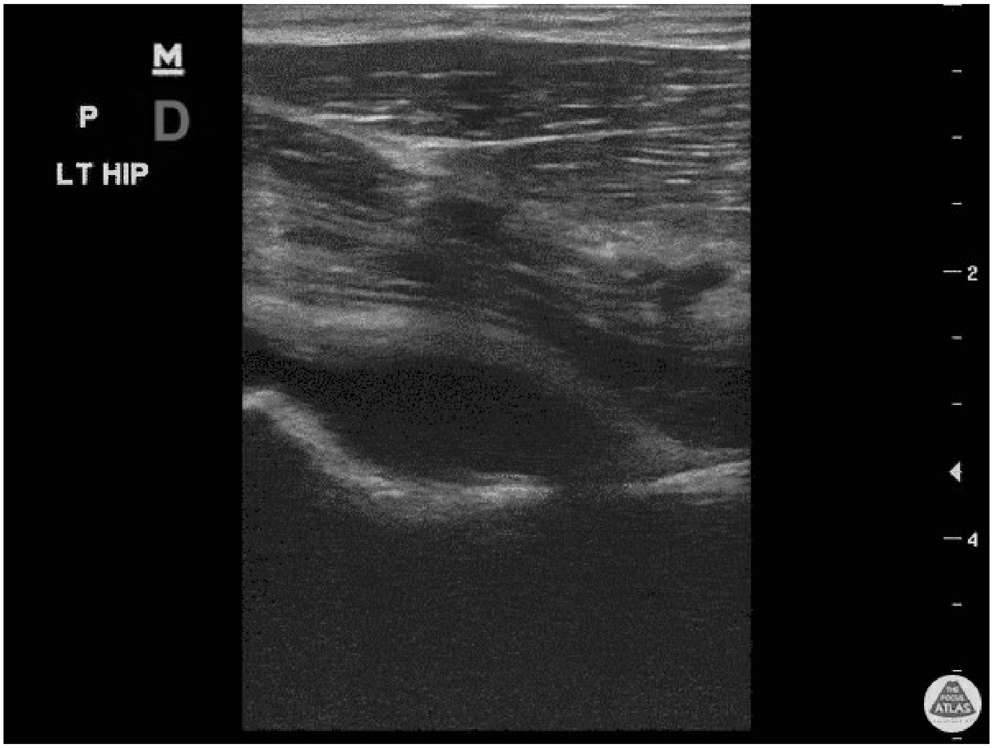
M
P
D
LT HIP
2
4

* 10. False positives of fractures on ultrasound include the following,**except:**

- Growth plates
- Vascular channels
- Malposition of probe
- Anisotropy

For question 11 to 14, label each of the following:

(The probe is located above the supra-spinatus, scanning on the saggital plane. A linear high-frequency probe was used)

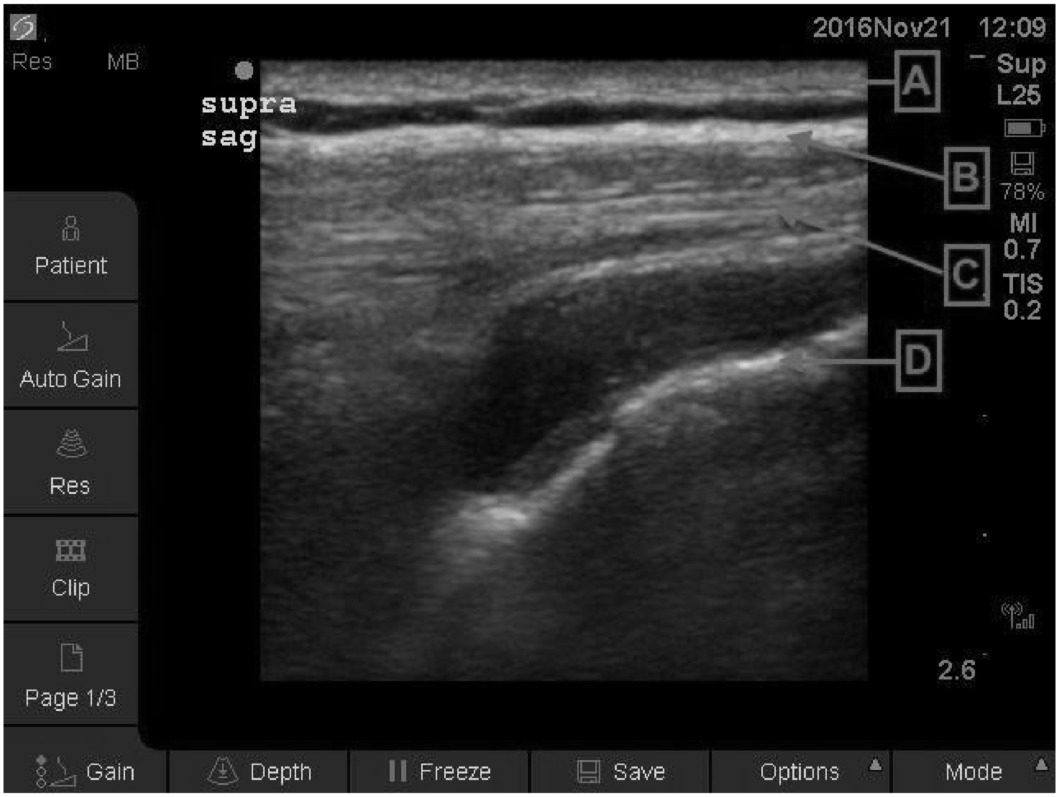

* 11. A

- Tendon
- Ligament
- Muscle
- Capsule
- Subcutaneous tissue
- Bone
- Fascial layer

* 12. B

- Tendon
- Ligament
- Muscle
- Capsule
- Subcutaneous tissue
- Bone
- Fascial layer

* 13. C

- Tendon
- Ligament
- Muscle
- Capsule
- Subcutaneous tissue
- Bone
- Fascial layer

* 14. D

- Tendon
- Ligament
- Muscle
- Capsule
- Subcutaneous tissue
- Bone
- Fascial layer

* 15. All the following findings are indicative of a complicated effusion,**except:**

- Anechoic fluid
- Septations within fluid
- Debris within fluid
- Irregular appearing collection

* 16. Identify which image demonstrates a joint effusion:

◯ A

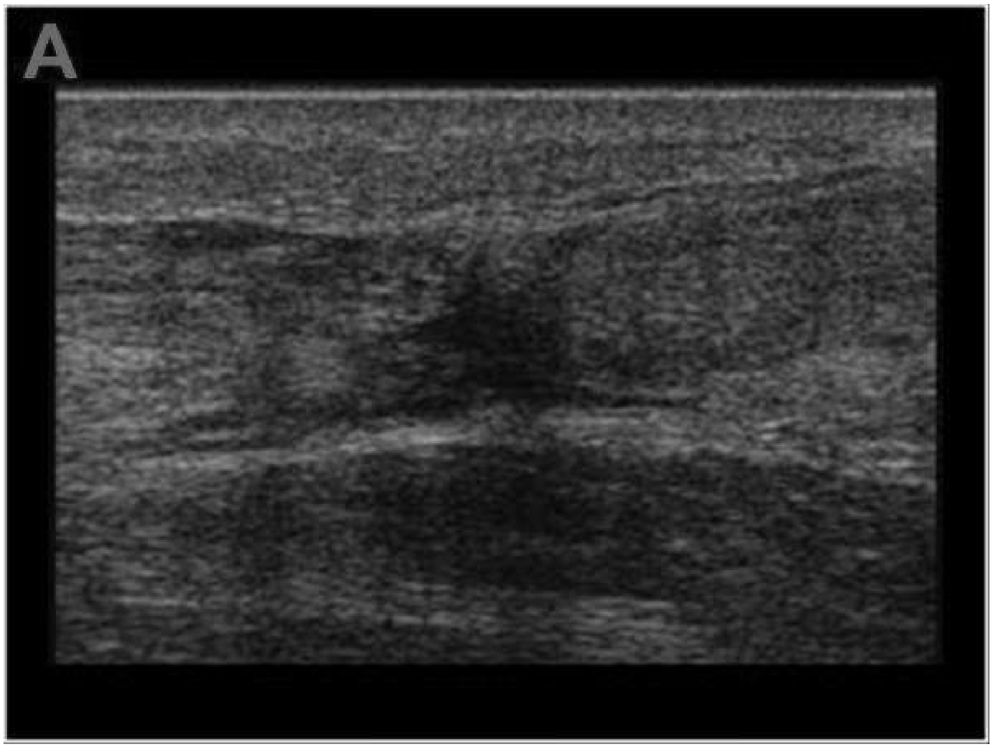

◯ B

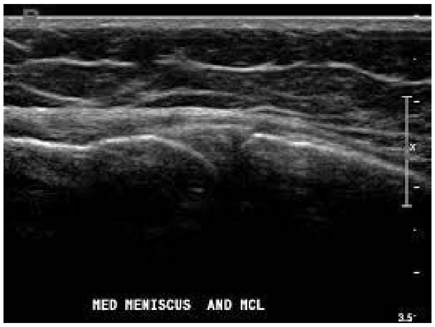

◯ C

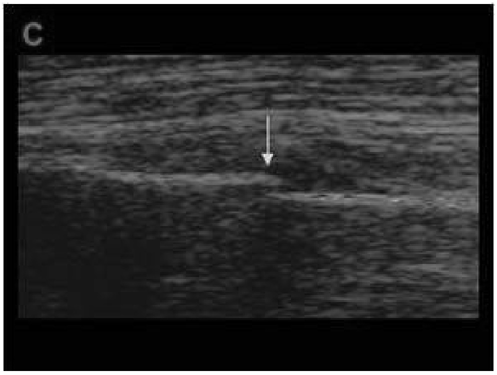

D

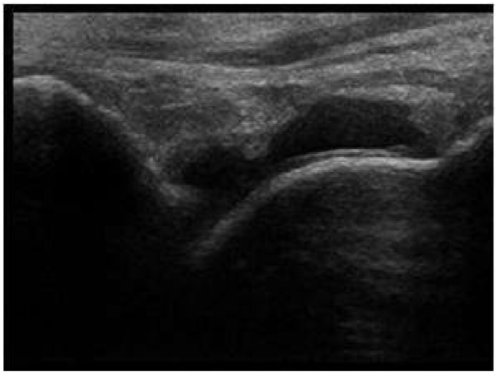

* 17. Identify which image demonstrates a joint dislocation:

A

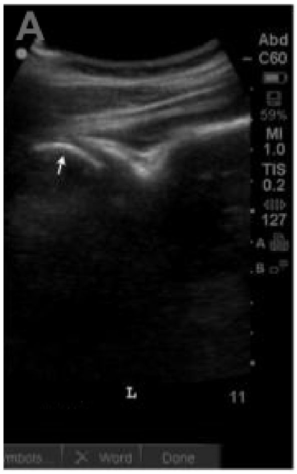

B

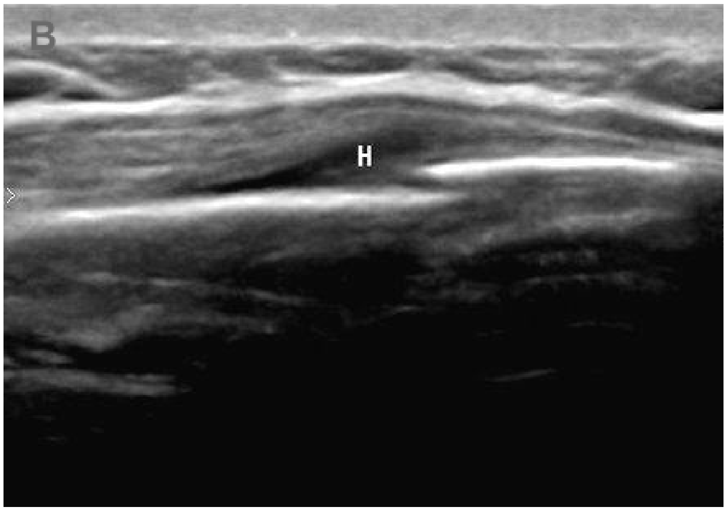

C

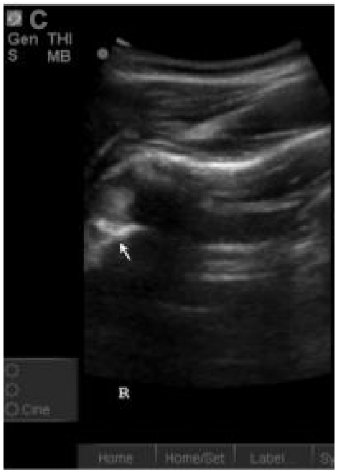

D

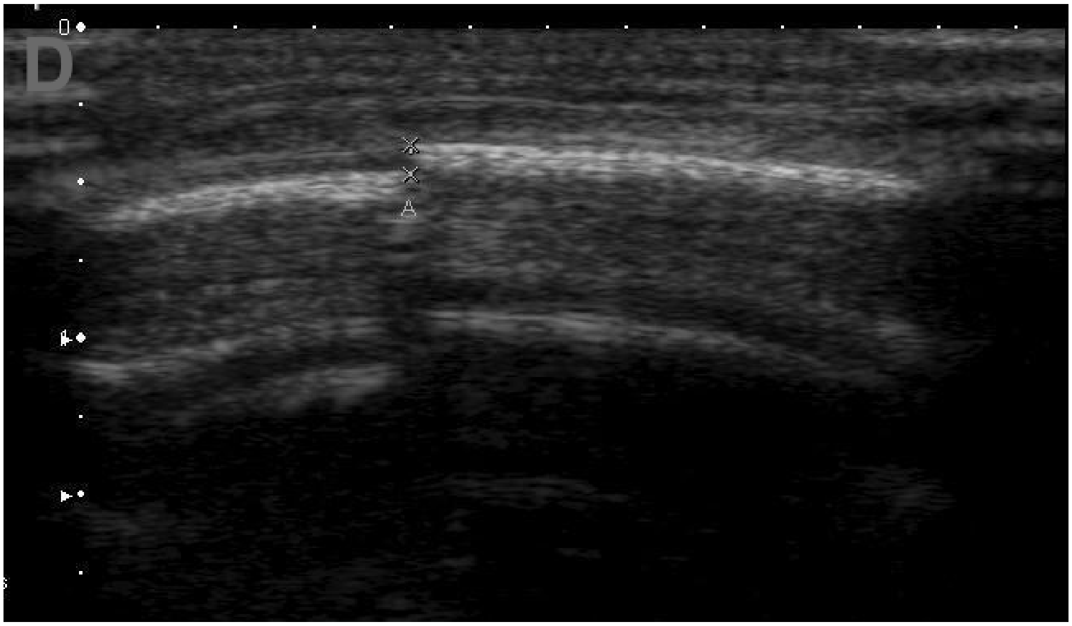

* 18. Identify which image contains a fracture:

- ◯ A

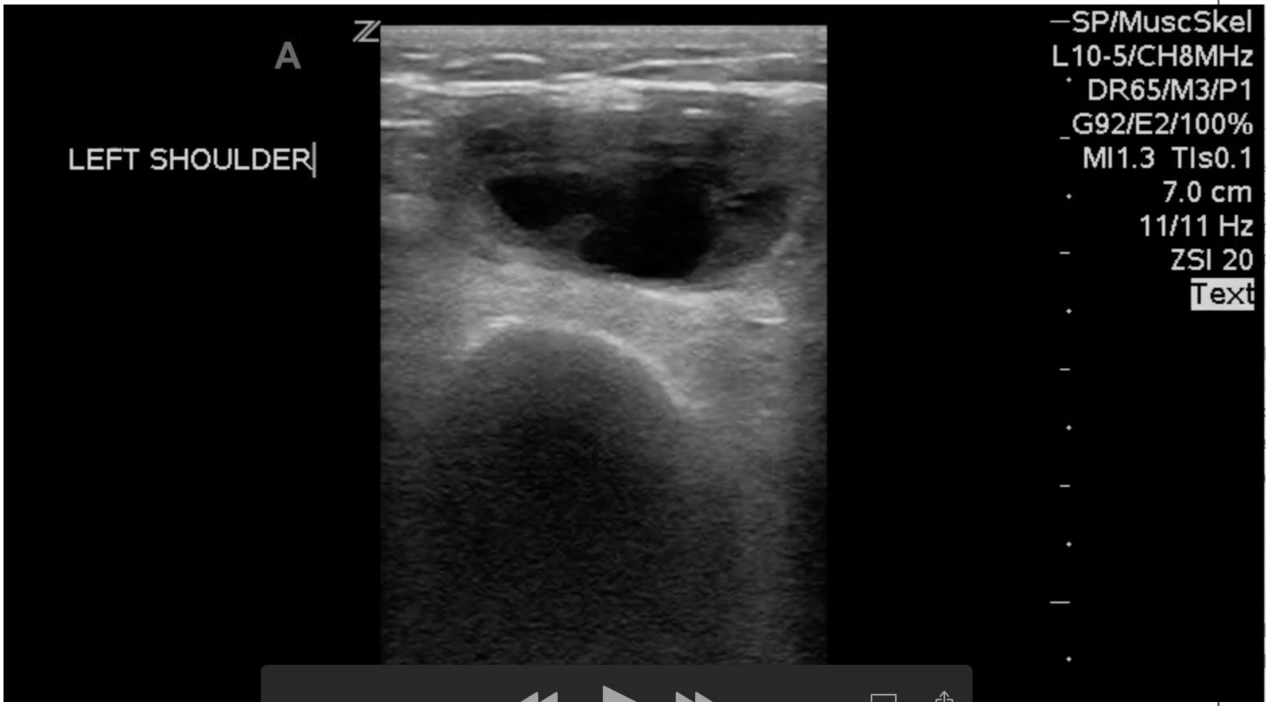

- ◯ B

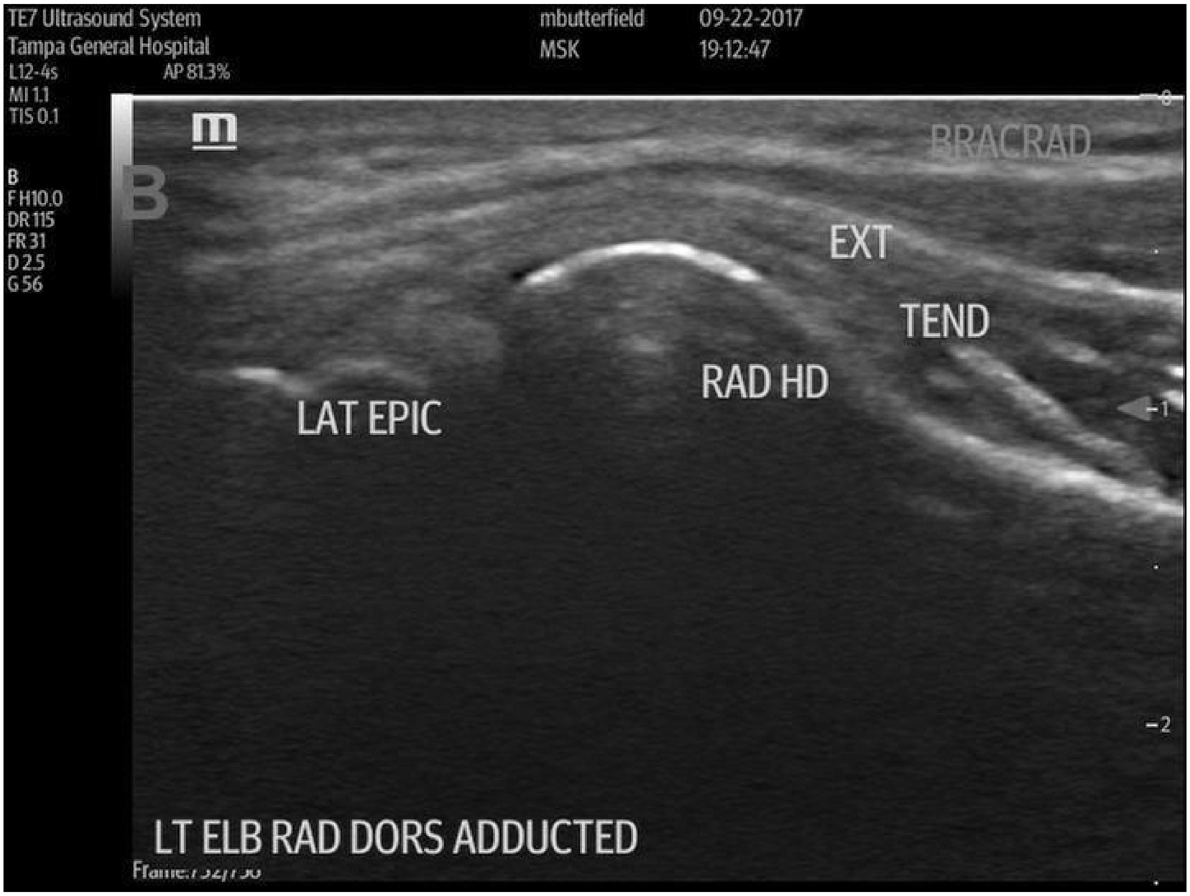

C

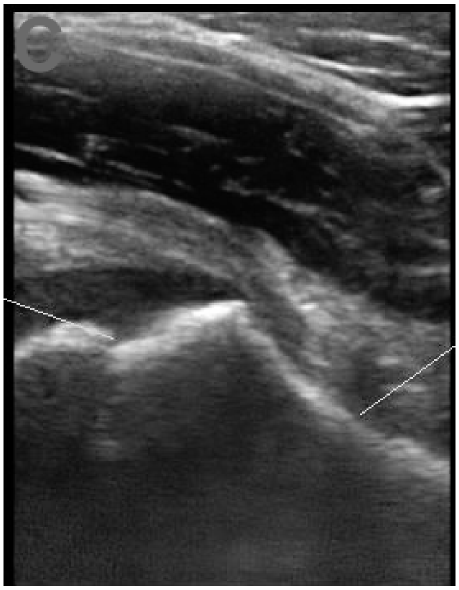

D

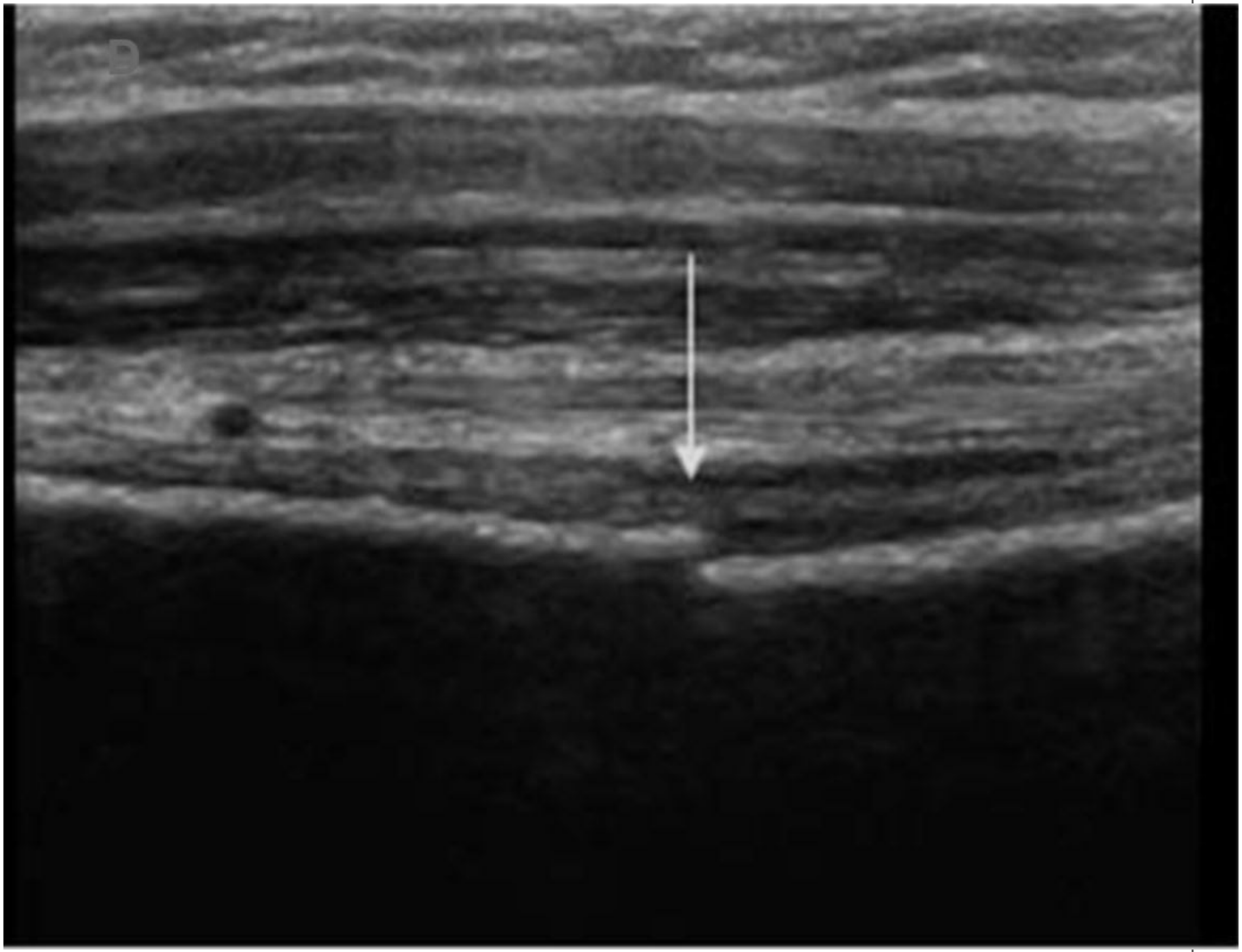

End of the test
