# Supplementary material for: Orthopaedic residents demonstrate retention of point of care ultrasound knowledge after a brief educational session: a quasi experimental study
Source: BMC Med Educ. 2019 Dec 30;19:474. doi: 10.1186/s12909-019-1916-0 (PMC6937626; doi:10.1186/s12909-019-1916-0)
Supplement: Supplementary file 3 — Additional file 3. Practical exam evaluation grid. The correction grid used by the evaluators to rate the participants’ performance while performing a diagnostic examination of the shoulder. [file 12909_2019_1916_MOESM3_ESM.pdf]

UNIVERSITY OF MANITOBA

Department of Surgery – Section of Orthopaedic Surgery
AD-420 720 McDermot Ave Winnipeg Manitoba R3E 0T3
P (204) 787-1219 F (204) 787-2460

# PRACTICAL EXAM EVALUATION GRID

**Study title:** Usefulness of a Musculoskeletal Ultrasound Course in an Orthopedic Surgery Residency Training program

**Participant code:** _____________

**Evaluation date:** DD / MM / 20 _ _

**Study visit:**

❑Pre-course ❑ Post-course ❑ 6 months ❑ 12 months

Correction key:

- Ask the resident to perform a shoulder examination as taught in the course, including setting up the ultrasound, examining the biceps tendon, supra-spinatus, infra-spinatus, subscapularis, sub-acromial bursae, and acromio-clavicular joint
- Give "**C**omplete" mark (2 points) when the step is completed perfectly, including all the movements described in the question stem, identifying the appropriate structure, without redirection from the examiner
- Give "**P**artial" mark (1 point) when the step is partially completed, or completed with redirection from the examiner. The appropriate structure must be identified
- Give "**I**nadequate" mark (0 point) if the step is not completed, or the wrong structure is identified.
- The order of the steps has no importance in the marking

| | Tasks | C | P | I |
|---|---|---|---|---|
| 1 | Setting up the ultrasound machine | | | |
| 1.1 | Positioning:<br>- Ensures patient's comfort, patient seated at appropriate height<br>- Ultrasound screen at adequate distance and in direct line of sight of the resident, without awkward rotation of the head<br>- Resident can stand in front of the patient or behind, as per personal preference. | | | |
| 1.2 | Selecting appropriate probe:<br>- High frequency linear array probe | | | |
| 1.3 | Adjusting depth:<br>- Depending on patient's build<br>- Should allow to see the full thickness of the tendons and part of underlying bone | | | |
| 1.4 | Adjusting gain:<br>- Depending on patient's build and echogenicity.<br>- Should be adequate to differentiate all the structures<br>- No inadequate noise to normally hypoechoic areas | | | |

UNIVERSITY OF MANITOBA

Department of Surgery – Section of Orthopaedic Surgery
AD-420 720 McDermot Ave Winnipeg Manitoba R3E 0T3
P (204) 787-1219   F (204) 787-2460

| 2 | Shoulder examination | C | P | I |
|---|---|---|---|---|
| 2.1 | Long head of biceps tendon<br>- Patient's hand resting on thigh, palm up, and elbow flexed 90°<br>- Short-axis from insertion to myotendinous junction<br>- Long-axis<br>- Tests for biceps tendon subluxation (external rotation) | | | |
| 2.2 | Subscapularis tendon:<br>- Patient externally rotates the arm with elbow flexed 90°<br>- Short-axis from insertion to myotendinous junction<br>- Long-axis | | | |
| 2.3 | Supraspinatus tendon:<br>- Patient puts the back of their hand to their back pocket<br>- Short-axis from insertion to myotendinous junction, must see anterior border<br>- Long-axis | | | |
| 2.4 | Subacromial bursae<br>- Same position<br>- Identifies the bursae: presence of fluid? thickened walls? | | | |
| 2.5 | Infraspinatus tendon<br>- Arm across the body, examined from behind the shoulder<br>- Short-axis from insertion to myotendinous junction<br>- Long-axis | | | |
| 2.6 | Gleno-humeral joint<br>- Arm in neutral position (hand on thigh), examined from behind<br>- Identifies the GH joint and presence/absence of fluid | | | |
| 2.7 | Acromio-clavicular joint<br>- Same position<br>- Identifies AC joint in coronal plane | | | |
| 2.8 | Subacromial impingement<br>- Same position, then followed by dynamic abduction of the shoulder<br>- Moves the transducer to the lateral edge of the acromion<br>- Identifies presence/absence of impingement | | | |
| | **Sub-total** (number of marks from each columns) | | | |
| | Multiplier | x2 | x1 | x0 |
| | Total for each column | | | |
| | **Final score** (max 24 points) | | | |
| | **Percent score** (score/24 X 100) | | | |

Evaluator signature: ______________________________  DD / MM / 20 _ _
