# Supplementary material for: Orthopaedic residents demonstrate retention of point of care ultrasound knowledge after a brief educational session: a quasi experimental study
Source: BMC Med Educ. 2019 Dec 30;19:474. doi: 10.1186/s12909-019-1916-0 (PMC6937626; doi:10.1186/s12909-019-1916-0)
Supplement: Supplementary file 4 — Additional file 4. Use of ultrasound in clinical setting. Printout of the online survey given to participants before the course and after 6 months. It evaluates their subjective comfort performing the different aspects of the MSK POCUS examination, as well as their frequency of use in the last 6 months. [file 12909_2019_1916_MOESM4_ESM.pdf]

UNIVERSITY OF MANITOBA

Musculoskeletal ultrasound - Use of ultrasound in clinical setting

Musculoskeletal ultrasound - Use in clinical setting

Thank you for participating in this research study. The goal is to assess of the effectiveness of a musculoskeletal ultrasound course in clinical education. Your answers to the following questions will help us have a better understanding of how and when you are using musculoskeletal ultrasound in clinic.
All answers are strictly confidential.

Version date: May 31st, 2017

* 1. Enter your participant number

* 2. Please indicate for which evaluation you are filling the survey:

- ○ First evaluation (before the course)
- ○ Six (6) months follow-up
- ○ Twelve (12) months follow-up

* 3. In the last six (6) months, how many times have you used ultrasound to perform the following tasks? (please answer to the best of your knowledge)

Diagnosing a ligament, tendon, muscle, nerve, or bone injury?

Performing a joint injection or aspiration?

Performing and injection or aspiration of any other region than a joint? (do not count placement of arterial or veinous lines)

Guiding the reduction of a fracture in the adult patient?

Guiding the reduction of pediatric fracture?

4. What is your comfort level in using the ultrasound for the following tasks (from 0 being very uncomfortable, to 10 being very comfortable)?

	1	2	3	4	5	6	7	8	9	10	N/A
Setting up the ultrasound machine?											
Choosing the appropriate probe?											
Adjusting the depth and contrast?											
Diagnosing a ligament, tendon, muscle, nerve, or bone injury?											
Performing a joint injection or aspiration?											
Performing an injection or aspiration in any other region than a joint? (do not count placement of arterial or veinous lines)											
Guiding the reduction of a fracture of the forearm?											
Guiding the reduction of any other fracture than the forearm?											
Performing ultrasound in general?											
